# Are envelope stress responses essential for persistence to β-lactams in *Escherichia coli*?

**DOI:** 10.1128/aac.00329-23

**Published:** 2023-10-03

**Authors:** Clothilde J. Rousseau, Nathan Fraikin, Safia Zedek, Laurence Van Melderen

**Affiliations:** 1 Bacterial Genetics and Physiology, Faculté des Sciences, Université Libre de Bruxelles (ULB), Gosselies, Belgium; Shionogi Inc., Florham Park, New Jersey, USA

**Keywords:** meropenem, σ^E^, Rcs

## Abstract

Bacterial persistence to antibiotics defines the ability of small sub-populations of sensitive cells within an isogenic population to survive high doses of bactericidal antibiotics. Here, we investigated the importance of the five main envelope stress responses (ESRs) of *Escherichia coli* in persistence to five bactericidal β-lactam antibiotics by combining classical time-kill curve experiments and single-cell analysis using time-lapse microscopy. We showed that the survival frequency of mutants for the Bae, Cpx, Psp, and Rcs systems treated with different β-lactams is comparable to that of the wild-type strain, indicating that these ESRs do not play a direct role in persistence to β-lactams. Since the σ^E^-encoding gene is essential, we could not directly test its role. Using fluorescent reporters to monitor the activation of ESRs, we observed that σ^E^ is induced by high doses of meropenem. However, the dynamics of σ^E^ activation during meropenem treatment did not reveal any difference in persister cells compared to the bulk of the population, indicating that σ^E^ activation is not a hallmark of persistence. The Bae, Cpx, Psp, and Rcs responses were neither induced by ampicillin nor by meropenem. However, pre-induction of the Rcs system by polymyxin B increased survival to meropenem in an Rcs-dependent manner, suggesting that this ESR might confer some yet uncharacterized advantages during meropenem treatment or at the post-antibiotic recovery step. Altogether, our data suggest that ESRs are not key actors in *E. coli* persistence to β-lactams in the conditions we tested.

## INTRODUCTION

Persistence to antibiotics is increasingly recognized as a cause of antibiotic treatment failure and emergence of resistance (1, 2). Persistence describes the ability of small sub-populations to survive high doses of bactericidal antibiotics independently of the acquisition of a resistance gene or mutation, instead being a consequence of cell-to-cell variations leading to phenotypic heterogeneity within a clonal population (3, 4). While resistance allows bacteria to actively grow in the presence of antibiotics and is represented by an increased minimal inhibitory concentration (MIC) value, persistence is a transient state that allows cells to tolerate the presence of antibiotics without a MIC increase and to resume growth after antibiotic removal. Persistence can be thought of as a phenotypic switch happening spontaneously or upon induction by antibiotics or other stress in a sub-population (5, 6). Spontaneous persistence is defined as appearing during steady-state growth, resulting in a small fraction of persister cells in the population under these conditions. The generation of persister cells upon external stress, like starvation during the stationary phase, is referred to as triggered persistence. Persister cells can be detected upon treatment at concentrations 10–100 times above the MIC, which induce the rapid killing of non-persister cells (5). Despite extensive studies, the molecular mechanisms underlying persistence remain poorly understood (7, 8). There is increasing evidence that persistence is a highly heterogeneous phenomenon that relies on different cellular events depending on the strain and the antibiotic (7, 9, 10). Instead of a universal path, persistence seems to be dependent on mechanisms linked to the mode of action of the antibiotic. For example, persister cell recovery from the DNA-damaging fluoroquinolones requires a functional SOS response, which is not required for survival to β-lactam antibiotics (11 – 13).

Beta-lactams inhibit the synthesis of the bacterial cell wall. More specifically, these antibiotics bind to penicillin-binding proteins (PBPs), a group of enzymes responsible for the synthesis, assembly, and recycling of peptidoglycan (PG) in the cell wall (14, 15). β-Lactams covalently and irreversibly bind PBPs, therefore compromising cell wall integrity. Without insertion of new PG units, proper cell wall synthesis is compromised, resulting in cell wall defects, sensitivity to osmotic pressure, and cell lysis. Modulation of envelope stress-related actors therefore appears to be a plausible survival strategy for persistence to β-lactams. A few studies indeed reported the induction of envelope stress responses (ESRs) by β-lactam antibiotics in *Escherichia coli* (16 – 19). ESRs monitor cell envelope integrity by responding to specific perturbations and activating the expression of a subset of genes whose products will allow a return to homeostasis. In *E. coli*, the five main ESRs are the Cpx, Bae, Rcs, Psp, and σ^E^ responses. The Cpx system is triggered by a broad range of stimuli (e.g*.,* misfolded proteins, metal ions, ethanol), but its main function is thought to be the sensing of misfolded periplasmic proteins and the induction of periplasmic chaperones and proteases (20, 21). The Bae ESR is induced by toxic compounds such as flavonoids, sodium tungstate, and ethanol. It controls the transcription of only a few genes, most of which are part of the Cpx regulon, such as the *mdt* genes encoding the MdtABC-TolC multidrug efflux pump, as well as its own transcription unit (22, 23). The Rcs system responds to outer membrane perturbations such as lipopolysaccharide (LPS) defects, osmotic shock, and growth on solid surfaces by regulating capsular synthesis, biofilm formation, and motility (24 – 27). The function of Psp, the phage-shock protein response, appears to be the restoration of the proton-motive force and is triggered by infection with the filamentous phage f1, ethanol exposure, heat shock, or inner membrane perturbations (28 – 30). Finally, the alternative sigma factor σ^E^ is the envelope heat-shock sigma factor. Unlike the ESR described above, σ^E^ is essential in *E. coli* K-12 strains (31). It is activated by the accumulation of unfolded outer membrane proteins (OMPs), which is triggered by heat shock, and also responds to alterations of the LPS structure (32). Activation of σ^E^ induces the expression of factors overseeing OMP transport, folding, and degradation and also inhibits their further production to restore outer membrane integrity (21, 32, 33). While activation of ESRs alters the transcriptome in different ways, their regulons overlap, and many envelope stressors trigger the activation of several pathways (e.g., ethanol induces the Bae, Cpx, Psp, and Rcs responses, indole induces the Bae and Rcs responses) (29, 34).

ESRs are thought to decrease susceptibility to several antimicrobial molecules through modulation of respiration to protect from antibiotic-induced ROS production as well as alteration of the cell surface by decreased porin production and increased efflux pump expression (35 – 37). Regarding *E. coli* susceptibility to β-lactams, mutants deleted for the Cpx or Rcs response regulators were shown to be more sensitive to mecillinam, and the Rcs mutant was also more sensitive to cefsulodin (17, 18). Moreover, activation of the Cpx response by overexpression of the NlpE OM lipoprotein and constitutive activation of the Rcs response in a phosphatase-deficient mutant were shown to decrease sensitivity to mecillinam (17, 18). Resistance to multiple β-lactams was also obtained by ectopic overexpression of the response regulators of the Bae, Cpx, and Rcs systems (38). In addition, transcriptomic study results hint that some ESRs can modulate cell wall reconstruction after PG damage, supporting the hypothesis that ESRs could indeed contribute to β-lactam survival. Notably, the Rcs system positively regulates expression of PBP1a- and PBP1b-encoding genes, two essential class A PBPs (24); the Cpx regulon includes *ldtD*, *ygaU*, and *slt*, which encode putative cell wall modification enzymes (39, 40).

Since different PG-related stressors and disruption of PBP activity by deletion of their encoding genes induce different ESRs (16), we hypothesized that modulation of ESR activity before or upon antibiotic exposure might lead to better survival and therefore trigger the persistence phenotype. Here, we show that mutants unable to elicit the Bae, Cpx, Rcs, or Psp responses are not affected for persistence to β-lactams in our experimental conditions. Using specific fluorescent transcriptional reporters, we observed that σ^E^ is the only ESR activated by meropenem. However, persister cells showed no difference in σ^E^ activity before and at early treatment times compared to dead cells, suggesting that σ^E^ is induced by antibiotic exposure but is not necessary for persistence. Pre-induction of Rcs by polymyxin B increased persistence to meropenem in an Rcs-dependent manner, indicating that this system might confer some advantages during meropenem treatment or during the recovery phase after the removal of the antibiotic.

## RESULTS

To characterize the potential role of ESRs in the survival to β-lactams, five bactericidal β-lactams were used (ampicillin, ceftazidime, ceftriaxone, meropenem, and ticarcillin). Polymyxin B, which targets the LPS and therefore disrupts the cell envelope independently of the poisoning of PBPs, was also included since it induces the Rcs response (41). Deletion mutants for the Bae, Cpx, Psp, and Rcs systems were constructed in the MG1655 laboratory strain. Since the σ^E^-encoding gene *rpoE* is essential in *E. coli* K-12 (31), we were unable to include this mutant in our analysis. We also constructed multiple deletion mutants since some ESRs were shown to be partially redundant, such as Bae and Cpx (22), or reported to be induced by high doses of ampicillin, such as Rcs and Psp (19). A mutant deleted for four ESRs (Bae^−^ Cpx^−^ Psp^−^ Rcs^−^) was constructed as well. Single and multiple deletions of the ESRs did not impact cell growth in steady-state conditions (Fig. S1).

### MIC and MBC of β-lactams are not affected in ESR mutants

The minimal inhibitory and bactericidal concentrations (MIC and MBC) were measured in liquid medium. The MIC value represents the minimal antibiotic dose required to prevent visible growth, i.e., an increase in turbidity in liquid medium, whereas the MBC value represents the minimal concentration that kills 99.9% of the cells after 24 h. The MBC value is therefore complementary to the MIC value for antibiotic susceptibility characterization. Since β-lactams are active on growing cells, MIC and MBC values were determined for *E. coli* exponentially growing cells (grown for about 4 h from stationary phase to an OD_600 nm_ of 0.3) in morpholinepropanesulfonic acid (MOPS) chemically defined medium supplemented with 0.4% glucose. ESR mutants show the same MICs and MBCs as the wild-type strain for the panel of antibiotics tested (Table 1). These observations suggest that ESRs do not play a major role in susceptibility to β-lactams and polymyxin B in the conditions tested. The MICs of the five β-lactams were also measured by the agar dilution method (42) on LA solid medium, and no appreciable difference was observed between the wild-type and mutant strains (Table S1).

**TABLE 1:** MIC and MBC determination for β-lactams and polymixin B of exponentially growing MG1655 and ESR deletion mutant strains in MOPS synthetic medium (µg/mL)^*a*^

Strain	AMP		CFZ		CTX		MER		TCR		Poly B	
	MIC	MBC	MIC	MBC	MIC	MBC	MIC	MBC	MIC	MBC	MIC	MBC
WT	8	16	8	8	2	2	1	1	4	16	4	4
Bae^−^	8	16	8	8	2	2	1	1	4	16	4	4
Cpx^−^	8	16	8	8	2	2	1	1	4	16	4	4
Psp^−^	8	16	8	8	2	2	1	1	4	16	4	4
Rcs^−^	8	16	8	8	2	2	1	1	4	16	4	4
Bae^−^ Cpx^−^	8	16	8	8	2	2	1	1	4	16	4	4
Psp^−^ Rcs^−^	8	16	8	8	2	2	1	1	4	16	4	4

^*a*^ *E. coli* MG1655 wild-type and ESR deletion mutants’ MICs and MBCs for ampicillin (AMP), ceftazidime (CFZ), ceftriaxone (CTX), meropenem (MER), ticarcillin (TCR), and polymyxin B (Poly B). MICs and MBCs were determined after 24 h of treatment. Measurements were performed at least three times.

### Survival to β-lactams is not affected in ESR mutants

**FIG 1:**
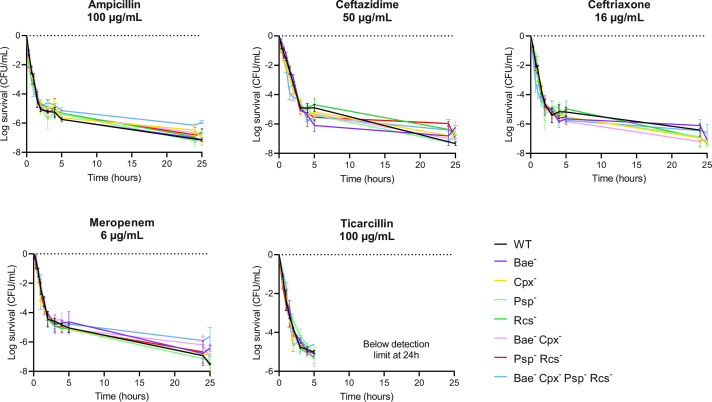
ESRs do not play a role in β-lactam survival. Time-kill curves of the wild-type and ESR mutant strains treated with β-lactams (ampicillin, ceftazidime, ceftriaxone, meropenem, ticarcillin). Cells grown to an OD_600 nm_ of 0.3 in MOPS medium were treated with sixfold the MBC of the corresponding antibiotic at time 0. The survival fraction was determined by plating diluted samples of the culture on LA plates at the indicated time points. Error bars represent the standard deviation from the mean of three independent experiments.

To evaluate the potential implication of ESRs in persistence, time-kill assays at high concentrations (sixfold the MBC) of bactericidal β-lactams (ampicillin, ceftazidime, ceftriaxone, meropenem, and ticarcillin) were performed. Survival to each antibiotic followed a typical biphasic curve, in which the first slope represents rapid killing of the vast majority of the population (non-persister cells), and the second slope reveals persister cells with slower killing dynamics. No appreciable difference in the killing phase or in the persister fraction was observed in the ESR mutants as compared to the wild-type strain, even for long-term treatment (24 h) (Fig. 1; Fig. S2). Altogether, these results show that ESRs neither contribute to the survival of the bulk of the population nor to persistence to β-lactams in the conditions tested.

### Ampicillin and meropenem treatments induce the σ^E^ response but not the other ESRs

**FIG 2:**
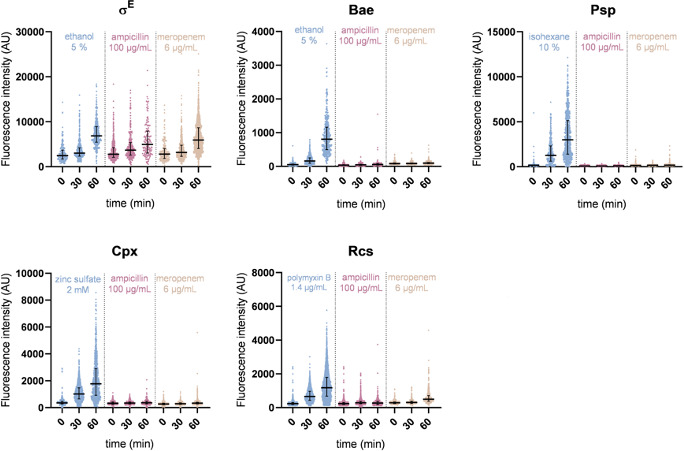
Ampicillin and meropenem induce the σ^E^ response but not the other ESRs. Distribution of the mean fluorescence of the ESR reporters over 60 min of treatment with the corresponding chemical inducer for each reporter (blue), ampicillin (100 µg/mL, pink), and meropenem (6 µg/mL, brown) recorded by fluorescence microscopy. Cells were grown in flasks to exponential phase in MOPS medium before treatment. For control conditions and meropenem treatment, samples were placed on agarose pads every 30 min for fluorescence microscopy picture acquisition. The same procedure was used for ampicillin treatment of the cells carrying the *p-rcsA*-mNG reporter, monitoring Rcs activation. For ampicillin treatment of cells carrying the other reporters, cells were introduced in a commercial microfluidic plate for time-lapse microscopy and left to grow for 3 h before ampicillin treatment. AU, arbitrary units. Bars represent the median with interquartile range.

Although ESR deletion mutants are not affected for survival or persistence to β-lactams , we tested whether some β-lactams were able to activate the ESRs. Importantly, this experiment allowed us to evaluate the potential contribution of σ^E^, as we could not test the corresponding mutant for the reason mentioned above. As ESR activity reporters, we constructed transcriptional fusions coupling the promoter of genes specifically regulated by the ESRs (Table 2) to the gene encoding the stable, fast-maturing fluorescent protein mNeonGreen (mNG). Reporters were validated by flow cytometry using specific inducers as positive controls (ethanol, zinc sulfate, isohexane, and polymyxin B, Table S2) in the wild-type strain and in the corresponding ESR mutants. All the ESR reporters were significantly induced by one or several stressors as indicated in the literature (29, 41, 43 – 45), and activation was abolished in the corresponding ESR mutant (Fig. S3a), showing that the transcriptional fusions are functional and report the activation of the corresponding ESR. Activation of the ESR reporters was monitored in exponentially growing wild-type cells over 60 min of treatment with ampicillin or meropenem, two β-lactams of different specificities, by fluorescence microscopy (see Materials and Methods for the procedure and controls). An additional control experiment was performed to ensure that expression of a fluorescent protein is not impacted by antibiotic exposure (Fig. S3b). For the Bae, Cpx, Psp, and Rcs reporters, no or only marginal induction was measured for both antibiotics as compared to the stressor-positive controls (Table 2; Fig. S3a). A 2.1-fold activation of the σ^E^ reporter, comparable to the stressor control (2.8-fold), was observed in the presence of meropenem. A slight induction was observed in the presence of ampicillin (1.6-fold) (Fig. 2; Table 2; Fig. S3a). Altogether, these data indicate that σ^E^ is the only tested ESR that is induced in response to meropenem and, to a lesser extent, ampicillin, in the conditions we used.

**TABLE 2:** ESR reporter activity during ampicillin and meropenem treatment^*a*^

Reporter gene	ESR	Control		Amp 100 µg/mL	Mpn 6 µg/mL
		Inducer	Fold-change	Fold-change	Fold-change
*micA*	σ^E^	Ethanol	2.8	1.8	2.4
*spy*	Bae	Ethanol	15.0	1.5	1.2
*pspA*	Psp	Isohexane	18.3	1.1	1.2
*cpxP*	Cpx	Zinc sulfate	5.0	1.1	1.2
*rcsA*	Rcs	Poly B	5.0	1.1	1.8
*pro1p*	*N.A*.	Ethanol	1.5	1.0	1.1
		Isohexane	1.3		
		Zinc sulfate	1.1		
		Poly B	1.1		

^*a*^ Activation of ESR-dependent fluorescent transcriptional reporters by 60-min treatment with positive controls (inducer) and ampicillin (Amp) at 100 µg/mL or meropenem (Mpn) at 6 µg/mL, measured by fluorescence microscopy. The reporter genes allowing monitoring of the induction of each ESR are indicated. The synthetic promoter *pro1p* (*pro1p*-mNG) was used as an internal control. Data extracted from Fig. 2; Fig. S6. Poly B, Polymyxin B.

### σ^E^ activity is not a meropenem persistence marker

Since σ^E^ is activated by meropenem, we focused on this ESR and assessed the dynamics of the response at the single-cell level using fluorescence time-lapse microscopy. If increased σ^E^ activity is linked to higher meropenem survival, this experiment will allow us to determine if persister cells emerge from cells that have high meropenem-induced σ^E^ activity (which would correspond to triggered persistence) or from cells with high σ^E^ levels before meropenem addition (spontaneous persistence). Cells were grown in the microfluidic chamber for 4 h and then treated for 3 h with meropenem. Twenty-five persister cells were observed among around 40,000 cells analyzed, corresponding to a 20-fold increase in persistence frequency as compared to that measured in cultures grown and treated in flasks (Fig. 1). Importantly, these 25 persister cells originated from cells that were growing and dividing before the addition of meropenem, indicating that they are not carry-over dormant cells from the previous stationary phase culture (Fig. S4).

**FIG 3:**
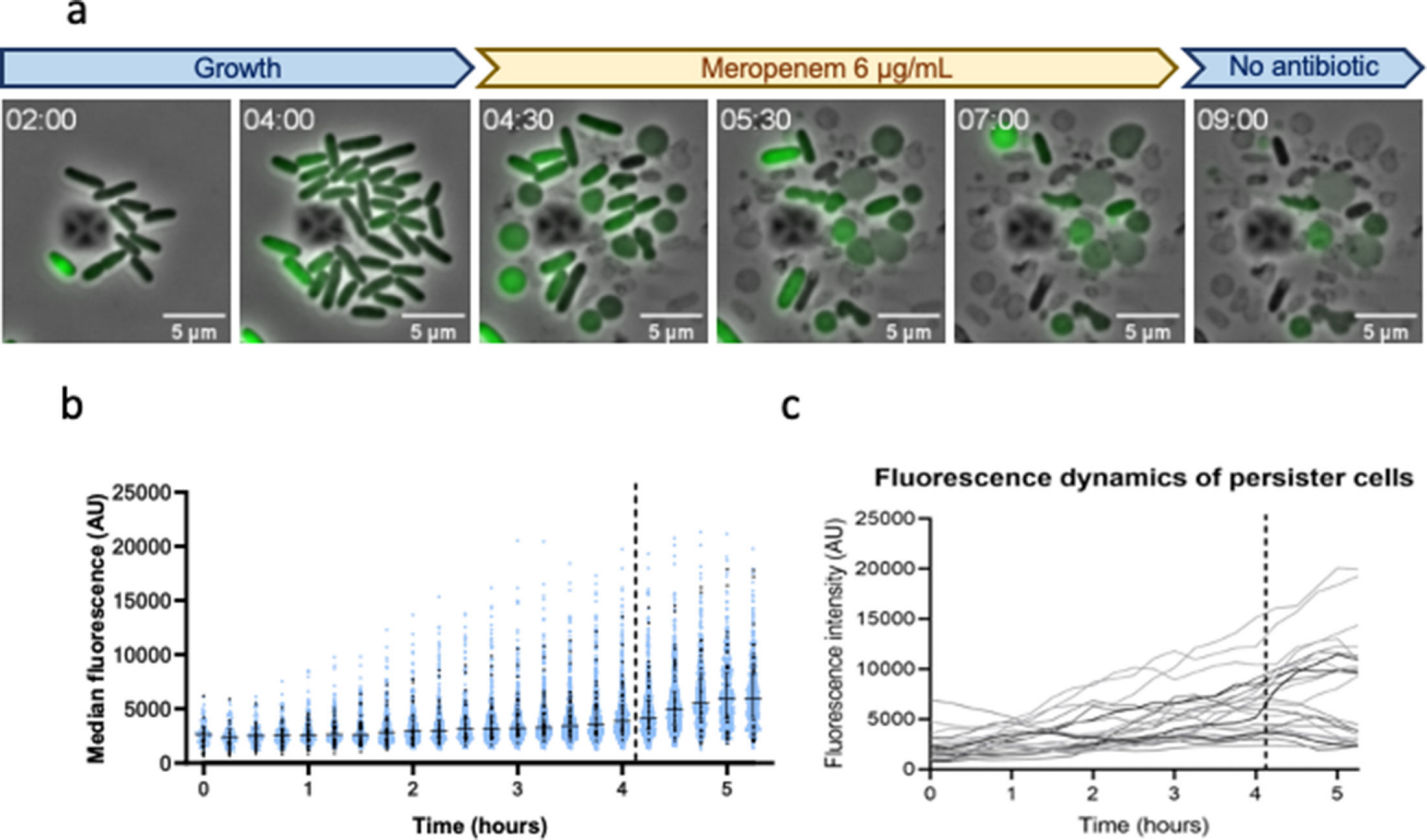
Single-cell imaging of σ^E^ activation during meropenem treatment. Cells carrying the p-*micA*-mNG fluorescent reporter monitoring σ^E^ activation were introduced in a commercial microfluidic plate for time-lapse microscopy and left to grow for 4 h before 3 h of meropenem treatment (6 µg/mL). (**a**) Imaging of non-persister cells at the indicated time (h). (**b**) σ^E^ fluorescence dynamics in non-surviving (blue dots) and surviving (black dots) cells. Bars represent the median with interquartile range. (**c**) σ^E^ fluorescence dynamics in persister cells. The median fluorescence of each persister cell plotted as a function of time. The dotted line indicates the start of meropenem treatment. AU, arbitrary units.

The σ^E^ reporter fluorescence signal was monitored in the population during growth before treatment and for the first hour of the meropenem treatment. Fluorescence could not be monitored for longer times as meropenem rapidly induces aberrant cell shapes and bursts, leading to loss of fluorescence and uninterpretable signals (Fig. 3a). Before and during the first hour of meropenem treatment, the fluorescence intensity was variable from cell to cell, and the median fluorescence increased with time, indicating that σ^E^ might be triggered by the microfluidic conditions (Fig. 3b), as previously reported for the Rcs system (46). Therefore, we could not distinguish between the potential effect of meropenem on σ^E^ activation and the effect of the experimental setup. The dynamics and extent of σ^E^ activation are variable from one persister cell to the other and follow those observed for non-persister cells (Fig. 3b and c), with about half of the persister cells showing no increase in fluorescence upon meropenem addition. From this experiment, we conclude that a high level of σ^E^ activation does not lead to meropenem persistence.

**FIG 4:**
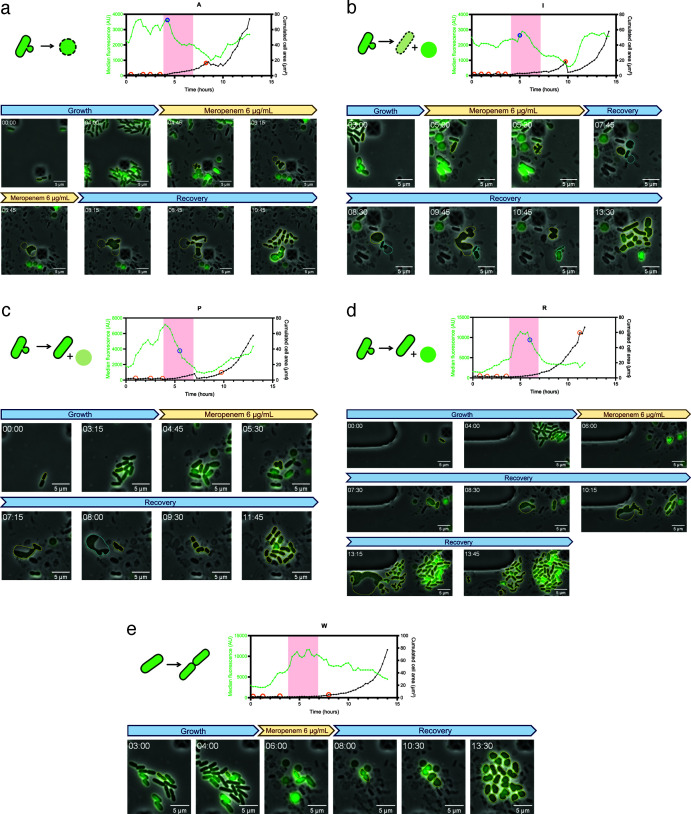
Persister cell morphology during meropenem treatment and recovery. Cells carrying the p-*micA*-mNG fluorescent reporter monitoring σ^E^ activation were introduced in a commercial microfluidic plate for time-lapse microscopy and left to grow for 4 h before 3 h of meropenem treatment (6 µg/mL). (**a–e**) Persister cells with different recovery behaviors. Yellow outlines indicate the surviving cells, and blue indicates the abortive daughter cells. Scale bars represent 5 µm. Illustrations indicate the recovery morphology and phenotype of each persister cell. The graphs above the pictures represent the persister cell median fluorescence and cumulated cell area observed in the microfluidic chamber plotted as a function of time. The red-shaded area indicates meropenem treatment. Orange circles indicate divisions during growth before treatment as well as the first division during recovery after treatment. The blue circle indicates the time point when the persister cell lost its rod shape.

Persister cells displayed different morphologies during meropenem treatment, as observed for the non-persister cells (Fig. 3a; Fig. 4; Fig. S4; Movie S1). During the recovery phase, persister cells divide by blebbing, vesiculation, and sometimes scission of a large cell into smaller ones. After blebbing, the daughter cell, which ultimately leads to the generation of viable cells with wild-type morphology, can be either rod-shaped or spheroplasts (examples of the different morphologies observed are shown in Fig. 4, and details of each persister cell’s dynamics are shown in Fig. S4). Some of the daughter cells were abortive, i.e., not able to give rise to viable cells (Fig. 4; Movie S1). Since some persister cells survived meropenem as spheroplasts (and divided only after antibiotic removal), the cell wall does not seem to be essential for survival to meropenem. Furthermore, some rod-shaped cells survived meropenem treatment, indicating that cell wall loss is not essential for meropenem survival. Unlike L-form bacteria, which are completely devoid of cell walls and therefore resistant to β-lactams (47), spheroplasting persister cells were not observed to divide during the meropenem treatment. It is unclear whether all the persister cells we observed retained some peptidoglycan, and we cannot exclude that some of them were real L-forms that would have divided during meropenem treatment if carried on for a longer time. Overall, we observed heterogeneity in cell morphology and σ^E^ activity in persister cells, which are similar for dying cells.

### Activation of Rcs prior to treatment increases survival to meropenem but not to ampicillin

**FIG 5:**
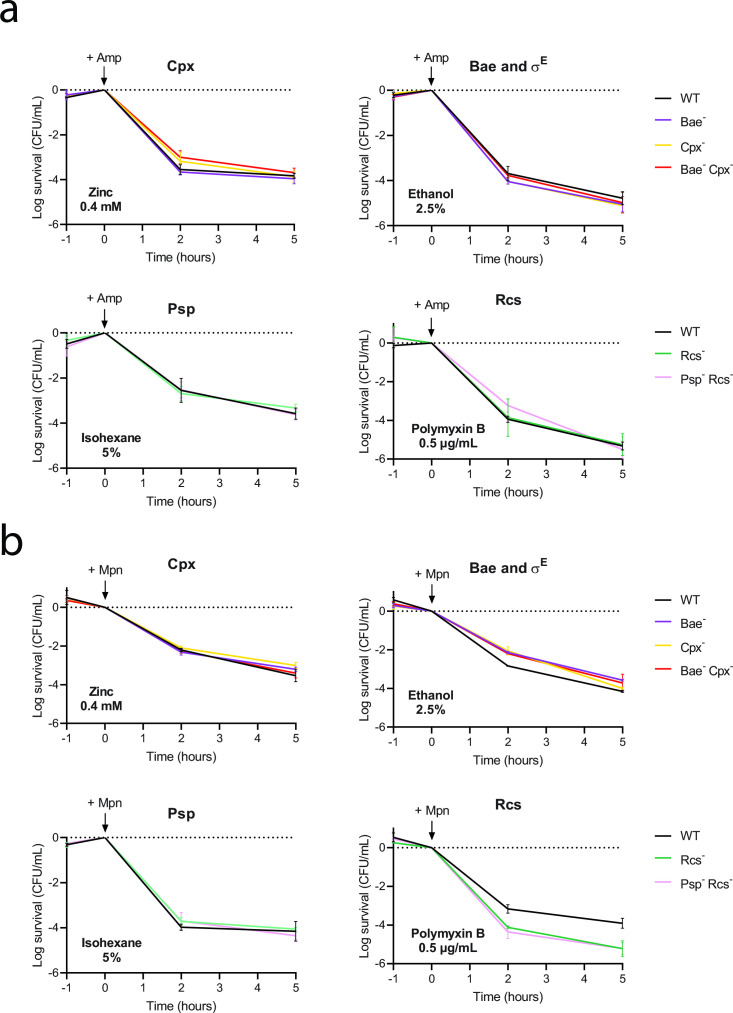
ESR chemical induction impact on ampicillin or meropenem killing. Time-kill curves of the wild-type and ESR mutant strains treated with (**a**) ampicillin and (**b**) meropenem after 1 h of ESR activation by chemical inducers. Cells grown to an OD_600 nm_ of 0.15 in MOPS medium were treated with 2.5% ethanol, 0.4 mM zinc sulfate, 5% isohexane, or 0.5 µg/mL of polymyxin B for 1 h prior to treatment with ampicillin (100 µg/mL) or meropenem (6 µg/mL). The time of antibiotic addition is indicated by an arrow. The survival fraction was determined by plating diluted samples of the culture on LA plates at the indicated time points. Error bars represent the standard deviation from the mean of three independent experiments.

Although we could neither detect or conclude about the ESR activation by meropenem or ampicillin nor observe a decreased survival of the quadruple ESR mutant treated with these antibiotics, we nevertheless tested whether pre-induction of the ESRs by external stressors triggers the persistence phenotype (5), therefore increasing the survival to these antibiotics. Inducers used as positive controls to validate the functionality of the ESR reporters (Table S2; Fig. S3a) were added for 1 h at concentrations sufficient to induce the corresponding systems (Fig. S5). Survival to ampicillin or meropenem after pre-treatment was increased as compared to the non-pre-treated conditions (1-log to 2-log difference after 5 h of antibiotic addition) (Fig. 5). This suggests that even though the inducers did not drastically impact growth (Fig. S5a and b), they allowed some tolerance to the antibiotic treatment. Nevertheless, survival of the corresponding ESR mutants to meropenem or ampicillin after ethanol (Bae and σ^E^), zinc sulfate (Cpx), or isohexane (Psp) pre-treatments did not differ from that of the wild-type strain (Fig. 5), suggesting that the pre-induction of Bae, Cpx, Psp, and σ^E^ does not confer ampicillin or meropenem tolerance and the increased survival is rather due to an indirect effect. Interestingly, Rcs activation by pre-treatment with polymyxin B increased survival to meropenem in the wild-type strain by around 2-log compared to the Rcs^−^ mutant and the Psp^−^ Rcs^−^ double mutant (Fig. 5b). Increased susceptibility of Rcs mutants to polymyxin B has been previously reported in other species (48, 49). However, in our conditions, the MIC for polymyxin B of the Rcs^−^ mutant (Table 1; Table S1) as well as its growth rate in the presence of 0.5 µg/mL polymyxin B (Fig. S5a) were similar to those of the wild-type strain. This result suggests that Rcs induction by polymyxin B in the wild-type strain confers increased persistence to meropenem. This phenotype is specific to meropenem and was not observed for ampicillin.

## DISCUSSION

Several pathways have been suggested to underlie bacterial persistence, including dormancy, modulation of ATP levels, and oxidative stress (50 – 53). Since β-lactams target enzymes involved in cell wall synthesis and remodeling, we hypothesized that modulation of ESRs might help cells to cope with antibiotic-induced damage. ESRs are well described as systems protecting the envelope against various intra- and extracellular stresses. However, data linking them to the cell wall and to β-lactam survival and resistance in *E. coli* are scarce (16).

Our data indicate that the Cpx-, Bae-, Rcs-, and Psp-defective mutants do not exhibit increased sensitivity to high doses of β-lactams (sixfold MBC) and polymyxin B using three different methods, i.e*.,* measurement of solid and liquid MIC values, MBC values, and killing kinetics. It was shown that increased activity of ESRs could protect cells from antibiotic killing (17, 18, 38). By chemically triggering ESRs, we observed that pre-induction of these responses does not promote ampicillin or meropenem survival in the wild-type strain as compared to ESR-defective mutants, except for the Rcs system, which increases survival to meropenem upon activation by polymyxin B. This is in accordance with the data obtained by Mahoney and Silhavy, where the Cpx-defective mutant does not exhibit increased sensitivity to ampicillin (36). We also show that ESRs are not induced by β-lactams in general, except for the essential sigma factor σ^E^, which shows induction upon meropenem and, to a lesser extent, ampicillin treatment. However, single-cell observations showed no difference in σ^E^ activity in cells succumbing to the meropenem treatment compared to persister cells, both exhibiting comparable heterogeneity in cell morphology and σ^E^ reporter signal. Activation of σ^E^ response by meropenem might be a consequence of antibiotic-induced stress but not be required to prevent cell death. However, we cannot exclude that modulation of the σ^E^ response might help cells cope with the meropenem treatment for a longer time or help in the post-antibiotic recovery step. Furthermore, the increase in survival in the microfluidic setup may be due to the induction of the Rcs response by surface contact (46), protecting the cells against meropenem lethality as observed when pre-inducing Rcs with polymyxin B. Induction of both σ^E^ and Rcs, as well as the reduced mechanical stress in the microfluidic setup compared to culture in a flask, may allow cell wall loss and stable spheroplasting in some cells by conferring protection from osmotic pressure and other insults to the membrane. In osmoprotective conditions, some bacteria are able to switch to the L-form state, defined as a stable state in which growth and division are possible despite the absence of a cell wall. L-forms of pathogenic *E. coli* were recently reported to naturally occur in the bladder of patients with urinary tract infection (54). Similar to persister cells, the population generated by L-form bacteria is genetically identical to the original population, even after reversion to the wild-type phenotype (47). However, unlike persister cells to β-lactams, L-forms are transiently resistant to cell wall-targeting antibiotics due to their lack of cell wall. All of the spheroplasting persister cells we observed regained a cell wall while dividing after antibiotic removal. However, we cannot exclude that these cells would have been able to divide in a cell wall-less state after several hours of meropenem treatment. Indeed, L-form generation time has been reported to be longer than wild-type and highly heterogeneous (47).

Discrepancies between previous studies and our results might be due to variations in strains and conditions; in references (17, 18), authors focus on bacteriostatic antibiotics and work in Lysogeny broth (LB) rich medium, while we used MOPS chemically defined medium, in which ESR activity in steady state can be different for several reasons, including osmolarity and growth rate. Furthermore, ESR involvement in *E. coli* antibiotic resistance has been observed using overexpression of OMPs on multicopy vectors or constitutive activation of the transcriptional regulators, both of which increase the MIC of several antibiotics (17, 38, 55). However, overexpression can activate the systems to a non-physiological degree and likely inhibit cell growth, which would have a significant impact on β-lactam susceptibility (18, 56, 57). By pre-treating cells with specific chemical stressors at concentrations that do not massively impact cell growth, we reasoned that induction of ESRs in these conditions might recapitulate physiological levels of ESR induction. While moderate activation of the Cpx system was indeed shown to increase the MIC value of the bacteriostatic β-lactams mecillinam and cephalexin (18), our results do not support this observation for bactericidal β-lactams in the *E. coli* strain and conditions used in this work. However, we observed that pre-induction of Rcs by polymyxin B increases survival at a high concentration of meropenem. The mechanism underlying this observation remains uncharacterized.

ESRs appear to have various roles in different bacteria. Deletion of the *rpoE* gene encoding the σ^E^ response is viable in *Salmonella enterica* sevovar typhimurium (*S*. Typhimurium) and uropathogenic *E. coli* (UPEC), but not in *E. coli* K-12. Interestingly, modulation of Bae and Cpx activities has an impact on β-lactam and aminoglycoside resistance in *S*. Typhimurium (58). These systems were shown to promote resistance through downregulation of OMPs in an *S*. Typhimurium mutant with an altered outer membrane proteome that was selected for its high β-lactam resistance (58). Indeed, inactivation of the Cpx or Bae responses in this resistant mutant restored sensitivity to three of the four β-lactams tested, as well as gentamycin and streptomycin (58). Furthermore, the virulence of pathogenic strains is impacted by ESR activity. Overactivation of the Cpx response by deletion of the *cpxA* gene or in the cpxA* mutant, both leading to the buildup of phosphorylated CpxR regulator, is detrimental to virulence in *S*. Typhimurium and UPECs, which also points to the fact that overactivation of ESRs leads to non-physiological levels of activity (59 – 61). Interestingly, a Cpx-deficient mutant of UPEC but not *S*. Typhimurium shows decreased virulence, indicating that ESR roles are varied among strains and species despite their conservation. The Psp response has also been shown to have a role in UPEC intramacrophage survival and *S*. Typhimurium virulence (62 – 64). While our results do not support a role for ESRs in the survival to β-lactams *per se*, the fact that these responses can affect virulence and the outcome of pathogenesis also suggests that they can affect how a pathogen responds to an antibiotic treatment in an infectious context. However, a potential role for ESRs in antibiotic survival in an infectious context remains to be investigated.

## MATERIALS AND METHODS

### Bacterial strains, plasmids, and growth conditions

Bacterial strains and plasmids used in this study are detailed in Tables S4 and S5 . Oligonucleotides used in this study are listed in Table S6. The single-copy pBeloBAC11 derivative pNF02-mNG plasmid used for ESR fluorescent reporters was constructed by amplification of the mNG-encoding gene from pNF06 (65) using BAC-for and BAC-rev primers, followed by ligation of this fragment in pNF02 at MluI and ApaLI sites (66). For the construction of ESR fluorescent reporters, promoters were cloned upstream of the mNeonGreen CDS in the pNF02 vector. Deletion mutants were constructed using the lambda-red method (67). Deletions and plasmid constructs are detailed in the supplementary material.

Experiments were performed in MOPS medium, originally described by Neidhardt, Bloch, and Smith (68). This medium was prepared as previously standardized in our laboratory with 0.4% D-glucose as a carbon source (69). Cells were grown at 37°C, and 15 µg/mL chloramphenicol was added to maintain pNF02-mNG derivatives encoding ESR fluorescent reporters in overnight cultures only and not in the cells that were challenged with β-lactams. Plating of colony-forming units was performed on lysogeny agar (LA; 0.5% yeast extract, 1.0% tryptone, 0.5% sodium chloride, 1.2% agar; Invitrogen cat. 22700041).

### Minimal inhibitory and bactericidal concentration assays

Minimal inhibitory concentrations (MIC) and minimal bactericidal concentrations (MBC) in liquid medium were determined by the addition of twofold increasing concentrations of antibiotics in exponentially growing cultures at an OD_600 nm_ of 0.3. A sample was taken before antibiotic addition and plated on LA plates for CFU measurement. After 24 h, the minimal concentration preventing visible growth (an OD_600 nm_ of equal or below 0.3) was considered the MIC. Tenfold dilutions of the treated cultures were then plated on LA plates for MBC measurement. On the next day, the MBC was determined as the lowest concentration of antibiotic killing at least 3 orders of magnitude as compared to the initial population, before the antibiotic treatment.

### Killing assays

Killing assays were performed as described in reference (69). Briefly, overnight cultures in MOPS were diluted to an OD_600 nm_ of 0.025 in fresh MOPS medium and grown to an OD_600 nm_ of 0.3. These exponentially growing cells were treated with the indicated concentration of antibiotic at time 0, when a first sample was taken for the CFU count in the initial population (corresponding to around 3 × 10^8^ CFU/mL). Samples were then taken at the indicated time points. Culture samples were not centrifuged since β-lactam-treated cells are highly susceptible to centrifugation. Culture samples were diluted in phosphate-buffered saline (PBS), and cells were plated on LA plates to be counted the next day. CFU were counted on plates corresponding at least to the first 10-fold dilution. Survival was calculated as the number of CFU at each time point divided by the number of CFU at time 0.

### Flow cytometry analysis

Exponentially growing cultures were diluted 30-fold in PBS to be analyzed by an Attune Nxt flow cytometer (Thermo Fisher Scientific). Population data were gathered and gated using Attune Nxt Software 2.7.0. The median mNeonGreen signal was measured with a 488-nm laser and a 530/30-nm emission filter.

### Microscopy

All conditions were imaged with an Axio Observer.Z1 (Zeiss, Germany) microscope equipped with a Colibri 7 LED system (Zeiss) and an ORCA-Flash 4.0 v2 camera (Hamamatsu). Green fluorescence was measured with a 514 LED and filter set 49011 (Chroma). Single-cell fluorescence signal was analyzed using the MicrobeJ suite for ImageJ.

Since the activity of the Rcs-regulated *rcsA* promoter is known to be induced by surface contact in microfluidic setups (46), cells carrying the p-*rcsA*-mNG were grown to exponential phase (an OD_600 nm_ of 0.3) in flasks and treated at time 0 with the indicated chemical for control conditions, meropenem, and ampicillin treatment. Samples were taken at time 0, 30 min, and 60 min and placed under agarose pads (1% agarose in PBS) to be imaged. This procedure was also used for treatment with meropenem for all reporters.

For microfluidic experiments, exponentially growing cells were trapped in a CellASIC ONIX B04A-03 microfluidic plate (Millipore, USA) and perfused with fresh medium for 4 h prior to the antibiotic challenge (100 µg/mL ampicillin or 6 µg/mL meropenem). After 3 h of treatment, fresh antibiotic-free medium was perfused again for 16 h to observe persister cell recovery. The experiment was performed at 37°C, and images were taken at 15-min intervals.

The synthetic promoter *pro1p* (*pro1p*-mNG) was used as an internal control (70) to allow discrimination between promoter activation and fluorescence accumulation due to continuous maturation of the mNG protein, which is not compensated by dilution in daughter cells upon growth arrest or slower growth. Activity of the *pro1p*-mNG control reporter remained stable during ampicillin treatment (median of 2,478 AU at *t*_0_ and 2,471 AU at *t*_60 min_; Fig. S6), indicating that any activity increase for the ESR reporters can be attributed to a specific induction and not simply to fluorescence accumulation. However, we observed that meropenem leads to a slight fluorescence accumulation as *pro1p*-mNG’s signal increases 1.2-fold over 1 h of treatment.

## AFTER EPUB

*[This article was published on 3 October 2023 with an error in the References section. The section was updated in the current version, posted on 18 October 2023.]*

## SUPPLEMENTAL MATERIAL

Supplemental file 1 aac.00329-23-s0001.pdf Supplementary figures, tables, legends, methods

Supplemental file 2 aac.00329-23-s0002.mp4 Movie S1. Persister cells before, during, and after meropenem treatment. Cells carrying the p-*micA*-mNG fluorescent reporter monitoring σ^E^ activation were introduced in a commercial microfluidic plate for time-lapse microscopy and left to grow for 4 h before a 3-h meropenem treatment (6 µg/mL). Yellow outlines indicate the surviving cells, and blue indicates the abortive daughter cells. Scale bars represent 5 µm. The persister cell shown here is referred to as “I” in Fig. 4b & Fig. S4.

ASM does not own the copyrights to Supplemental Material that may be linked to, or accessed through, an article. The authors have granted ASM a non-exclusive, world-wide license to publish the Supplemental Material files. Please contact the corresponding author directly for reuse.
