# Supplementary material for: Are envelope stress responses essential for persistence to β-lactams in Escherichia coli?
Source: Antimicrob Agents Chemother. 2023 Oct 3;67(10):e00329-23. doi: 10.1128/aac.00329-23 (PMC10583663; doi:10.1128/aac.00329-23)
Supplement: Supplemental file 1 — Supplementary figures, tables, legends, methods [file aac.00329-23-s0001.pdf]

Supplementary Figures :

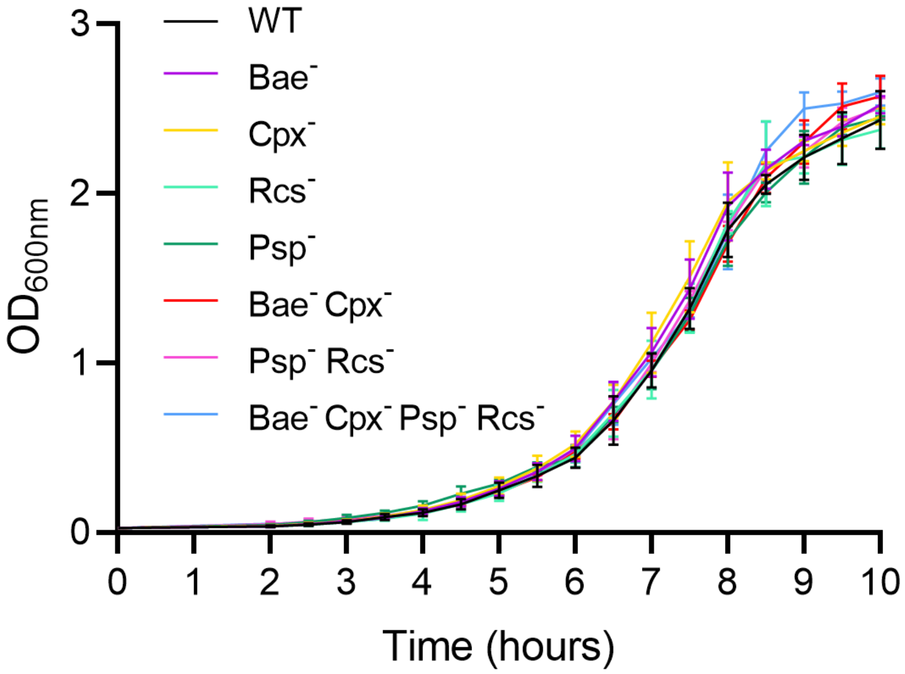

Figure S1. Growth curves of ESR mutants.

Growth curves of wild-type and ESRs mutant strains in MOPS medium measured by OD_{600nm}. Error bars represent standard deviation from the mean of three independent experiments.

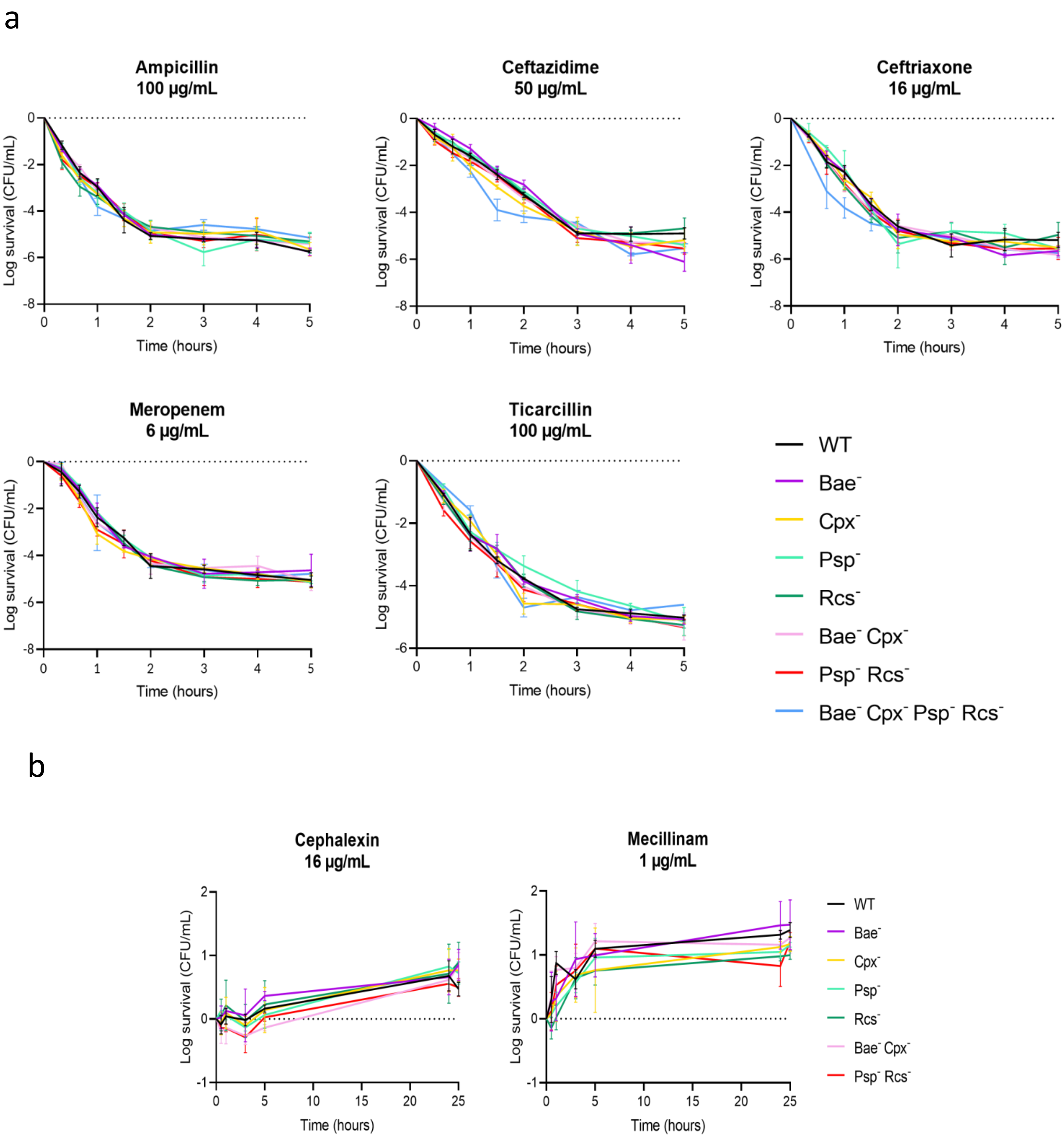

Figure S2. Killing dynamics to bactericidal and bacteriostatic β-lactams.

Survival kinetics of wild-type and ESRs mutants. **(a)** Biphasic killing curves to six times the MBC of different bactericidal β-lactams during the first 5 hours of treatment. **(b)** Survival to four times the MIC of bacteriostatic β-lactams cephalexin and mecillinam. Cells grown to OD_{600nm} 0.3 in MOPS medium were treated with for the corresponding antibiotic at time zero. The survival fraction was determined by plating diluted samples of the culture on LB agar plates at given times points. Error bars represent standard deviation from the mean of three independent experiments.

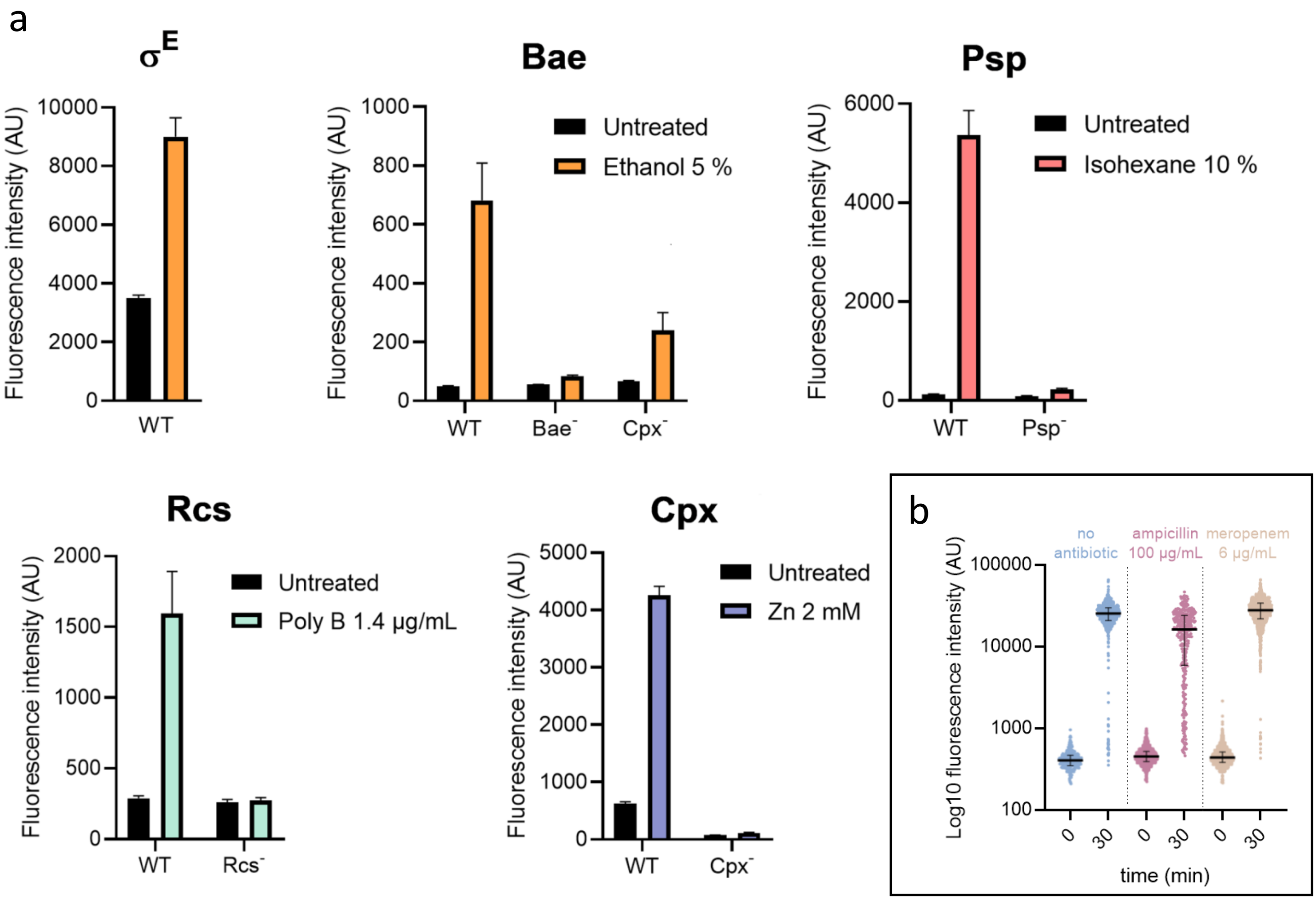

Figure S3. Validation of ESR reporters and fluorescence acquisition during antibiotic treatment

(a) Activity of transcriptional fusions analyzed by flow cytometry under stress conditions. Wild-type MG1655 and ESR mutants carrying fluorescent reporters were grown in MOPS medium to OD_{600nm} of 0.3 and treated during one hour with an inducing chemical. 2 mM zinc sulfate (purple), 5% ethanol (orange), 10% isohexane (pink), 1.4 µg/mL polymyxin B (green) of left untreated (black) before flow cytometry measurements. Data represent the mean of three independent experiment where the median fluorescence signal of at least 5000 events was recorded. Bars show standard deviation. **(b)** Validation of fluorescent protein maturation during antibiotic treatment. Wild-type cells carrying the pET-GFP plasmid with an IPTG-inducible GFP were grown in flask in MOPS medium to OD_{600nm} of 0.3. At t_0, ampicillin, meropenem, or no antibiotic was added at the same time as IPTG. Samples were placed under agarose pads for fluorescence microscopy pictures. Bars represent the median with interquartile range.

A.U., arbitrary units.

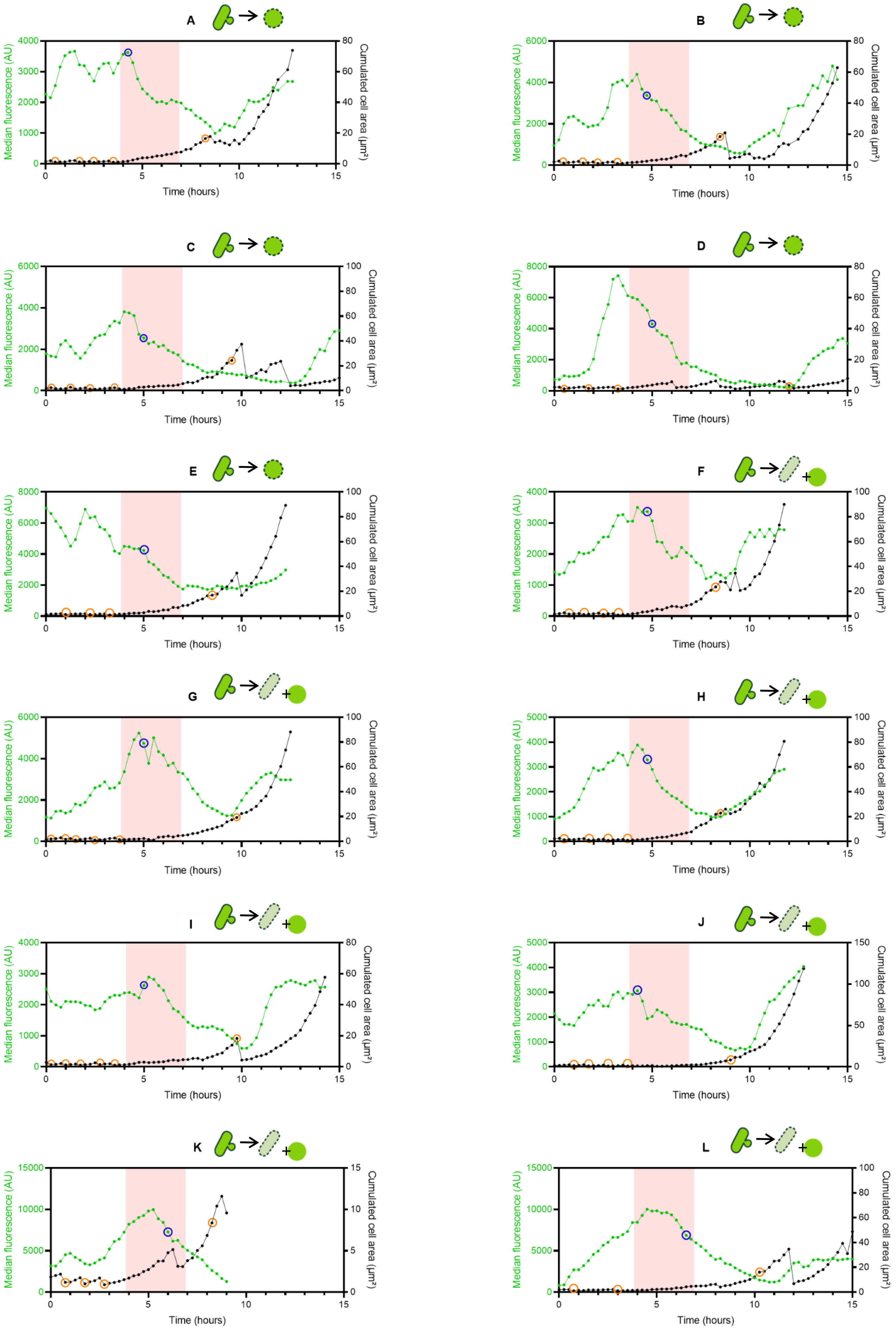
A
Median fluorescence (AU)
0
1000
2000
3000
4000
Cumulated cell area (µm²)
0
20
40
60
80
Time (hours)
0
5
10
15
B
Median fluorescence (AU)
0
2000
4000
6000
Cumulated cell area (µm²)
0
20
40
60
80
Time (hours)
0
5
10
15
C
Median fluorescence (AU)
0
2000
4000
6000
Cumulated cell area (µm²)
0
20
40
60
80
100
Time (hours)
0
5
10
15
D
Median fluorescence (AU)
0
2000
4000
6000
8000
Cumulated cell area (µm²)
0
20
40
60
80
Time (hours)
0
5
10
15
E
Median fluorescence (AU)
0
2000
4000
6000
8000
Cumulated cell area (µm²)
0
20
40
60
80
100
Time (hours)
0
5
10
15
F
Median fluorescence (AU)
0
1000
2000
3000
4000
Cumulated cell area (µm²)
0
20
40
60
80
100
Time (hours)
0
5
10
15
G
Median fluorescence (AU)
0
2000
4000
6000
Cumulated cell area (µm²)
0
20
40
60
80
100
Time (hours)
0
5
10
15
H
Median fluorescence (AU)
0
1000
2000
3000
4000
5000
Cumulated cell area (µm²)
0
20
40
60
80
100
Time (hours)
0
5
10
15
I
Median fluorescence (AU)
0
1000
2000
3000
4000
Cumulated cell area (µm²)
0
20
40
60
80
Time (hours)
0
5
10
15
J
Median fluorescence (AU)
0
1000
2000
3000
4000
5000
Cumulated cell area (µm²)
0
50
100
150
Time (hours)
0
5
10
15
K
Median fluorescence (AU)
0
5000
10000
15000
Cumulated cell area (µm²)
0
5
10
15
Time (hours)
0
5
10
15
L
Median fluorescence (AU)
0
5000
10000
15000
Cumulated cell area (µm²)
0
20
40
60
80
100
Time (hours)
0
5
10
15

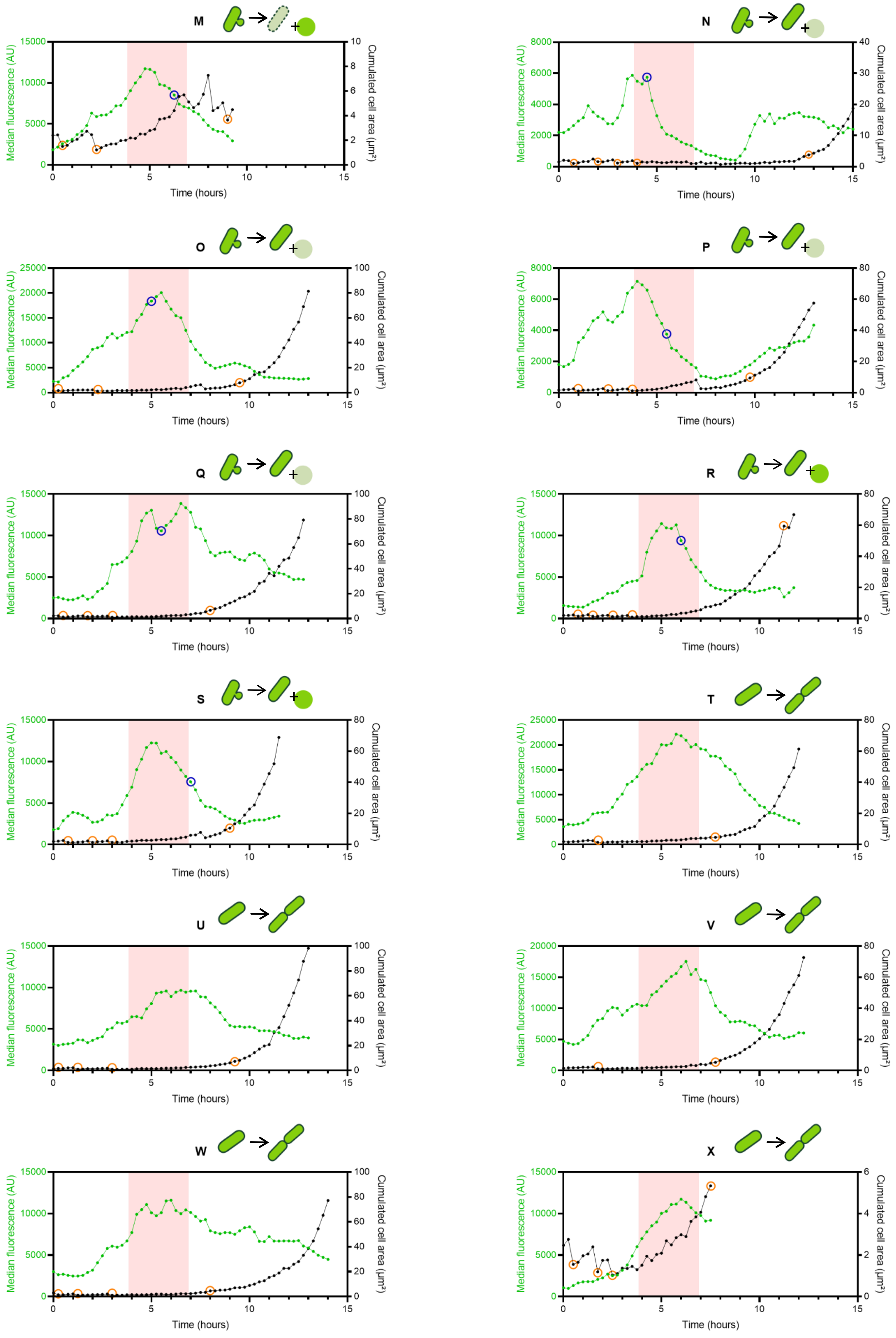
M
N
O
P
Q
R
S
T
U
V
W
X
Median fluorescence (AU)
Cumulated cell area (µm²)
Time (hours)

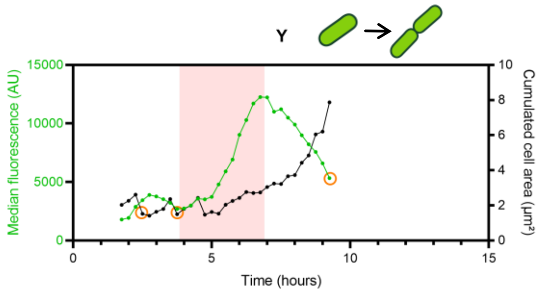

Figure S4. Individual σ^E fluorescent reporter activity and growth of the 25 persister cells before, during and after meropenem treatment

Cells carrying the p-*micA*-mNG fluorescent reporter monitoring σ^E activation were introduced in a commercial microfluidic plate for time-lapse microscopy and left to grow for 4 hours before 3-hours meropenem treatment (6 mg/mL). Median fluorescence and cumulated cell area of each persister cell observed in the microfluidic chamber plotted as a function of time. Increase in cell size during treatment is due to spheroplasting after cell wall loss. Red shaded area indicates meropenem treatment (6 µg/mL).

Orange circles indicate divisions during growth before treatment as well as the first division during recovery after treatment. Blue circle indicates the time point when the persister cell lost its rod shape.

Illustrations indicate the recovery morphology phenotype of each persister cell as described in Fig. 4.

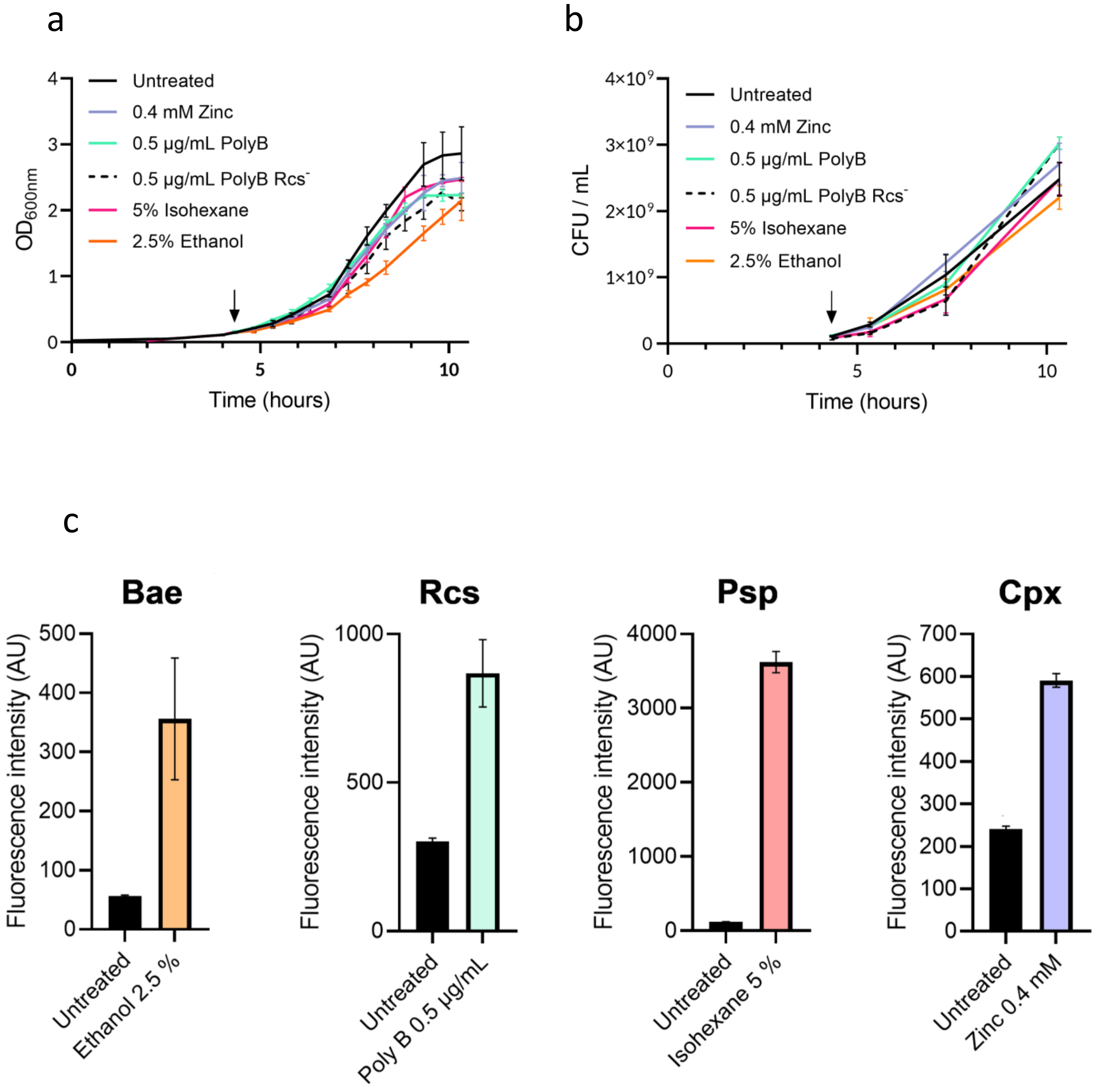

Figure S5. Validation of inducing conditions for pre-treatment

(a, b) Growth curves of wild-type cells in MOPS measured by OD_{600nm} **(a)** and CFU counts **(b)**. Once OD_{600nm} 0.15 was reached (indicated by arrow), 0.4 mM zinc sulfate (purple), 0.5 µg/mL Polymyxin B in WT (green), and in the Rcs^- mutant (dotted lines) 5 % isohexane (pink) or 2.5 % ethanol (orange) was added, or cells were left untreated (black). Error bars represent standard deviation from the mean of three independent experiments.

(c) Wild-type cells carrying fluorescent reporters were grown in MOPS medium to OD_{600nm} of 0.3 and treated during one hour with 0.4 mM zinc sulfate (purple), 2.5 % ethanol (orange), 5 % isohexane (pink), 0.5 µg/mL polymyxine B (green) or left untreated (black) before flow cytometry measurements. A.U., arbitrary units. Data represent the mean of three independent experiment where the median fluorescence signal of at least 5,000 events was recorded. Bars show standard deviation from the mean of three independent experiments.

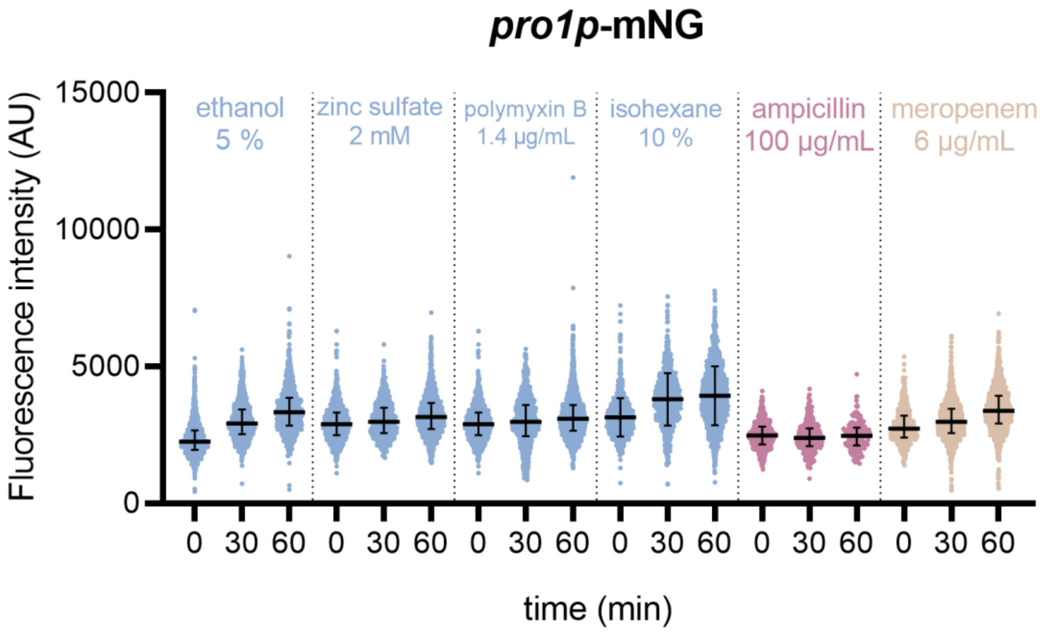

Figure S6. Fluorescence accumulation in different conditions

Distribution of mean fluorescence of single cells over one hour of treatment with the different chemicals used for reporter validation (blue), ampicillin (100 µg/mL, pink), and meropenem (6 µg/mL, brown) recorded by microscopy. Wild-type cells carrying a fusion of the insulated and constitutive *pro1p* synthetic promoter (Davis *et al.*, 2011) with mNeonGreen (*pro1p*-mNG) were grown in flask in MOPS medium to OD_{600nm} of 0.3 before treatment. Samples were placed under agarose pads for fluorescence microscopy pictures. A.U., arbitrary units. Bars represent the median with interquartile range.

<u>Supplementary movies :</u>

Movie S1. Persister cells before, during and after meropenem treatment

Cells carrying the p-*micA*-mNG fluorescent reporter monitoring σ^E activation were introduced in a commercial microfluidic plate for time-lapse microscopy and left to grow for 4 hours before 3-hours meropenem treatment (6 mg/mL). Yellow outlines indicate the surviving cells, blue indicates the abortive daughter cells. Scale bars represent 5 µm. The persister cell shown here is referred to as « I » in Fig 4b and S4.

Supplementary tables

Table S1: MIC determination by the agar dilution method

Strain	AMP	CFZ	CTX	MER	TCR	Poly B
WT	4	0.25	0.0125	0.025	2	0.25
Bae⁻	4	0.25	0.0125	0.025	2	0.25
Cpx⁻	4	0.25	0.0125	0.015	2	0.25
Psp⁻	4	0.25	0.0125	0.025	2	0.25
Rcs⁻	4	0.25	0.0125	0.015	2	0.25
Bae⁻ Cpx⁻	4	0.25	0.0125	0.025	2	0.25
Psp⁻ Rcs⁻	4	0.25	0.0125	0.015	2	0.25
Bae⁻ Cpx⁻ Psp⁻ Rcs⁻	2	0.125	0.00625	0.025	1	ND

Minimal inhibitory concentrations (MICs) of ampicillin (AMP), ceftazidime (CFZ), ceftriaxone (CTX), meropenem (MER), ticarcillin (TCR) and polymyxin B (Poly B) measured as µg/ml for the wild-type and ESR mutant strains by the agar dilution method (1). Measurements were done at least 3 times.

Table S2: ESR reporters and activation conditions

Reporter gene	ESR	Inducer	Concentration used		Reference
			Reporter validation	Pre-induction	
spy	Bae	Ethanol	5%	2.5%	(2)
cpxP	Cpx	Zinc Sulfate	2 mM	0.4 mM	(3)
pspA	Psp	Isohexane	10%	5%	(4)
rcsA	Rcs	Polymyxin B	1.4 µg/mL	0.5 µg/mL	(5)
micA	σ^E	Ethanol	5%	2.5%	(6)
pro1p	N.A.	Fluorescence accumulation control	N.A.	N.A.	(7)

N.A. not applicable

Table S3: ESR reporter activity during treatment

Reporter gene	ESR	Control						Amp 100 µg/mL					Mpn 6 µg/mL				
		**Inducer**	**t0**	**IQR**	**t60min**	**IQR**	**Fold-change**	**t0**	**IQR**	**t60min**	**IQR**	**Fold-change**	**t0**	**IQR**	**t60min**	**IQR**	**Fold-change**
micA	**σ^E**	Ethanol	2,461	1,988	6,862	3,428	**2.8**	2,779	2,170	4,966	4,843	**1.8**	2,440	1,625	5,947	4,635	**2.4**
spy	**Bae**	Ethanol	53	33	809	658	**15.0**	39	28	60	41	**1.5**	84	30	99	40	**1.2**
pspA	**Psp**	Isohexane	164	66	3,009	3,734	**18.3**	84	46	95	73	**1.1**	146	55	181	78	**1.2**
cpxP	**Cpx**	Zinc	356	186	1,768	1,992	**5.0**	318	164	364	194	**1.1**	269	115	327	123	**1.2**
rcsA	**Rcs**	Poly B	238	117	1,175	1,112	**5.0**	238	117	267	182	**1.1**	282	98	496	322	**1.8**
pro1p	*N.A.*	Ethanol	2,256	712	3,333	1,011	**1.5**	2,478	641	2,471	649	**1.0**	2,569	699	2,827	1163	**1.1**
		Isohexane	3,081	815	3,897	1,426	**1.3**										
		Zinc	2,890	826	3,157	951	**1.1**										
		Poly B	2,890	826	3,092	934	**1.1**										

Data from Figure 2 and S6. Median fluorescence in A.U. (arbitrary units) before (t0) and after 60 min (t60min) of treatment with indicated chemical, and interquartile range (IQR). Amp: ampicillin, Mpn: meropenem, PolyB: polymyxin B. Inducer concentrations (control) for reporter validation are mentioned in Table S2.

Table S4: Strain list

Strain	Genotype	Reference
MG1655	*E. coli* K-12 F- λ- *ilvG rfb-50 rph-1*	(8)
Bae^-	MG1655 Δ*baeSR::FRT*	This study
Cpx^-	MG1655 Δ*cpxQPRA::FRT*	This study
Bae^- Cpx^-	MG1655 Δ*baeSR::FRT* Δ*cpxQPRA::FRT*	This study
Psp^-	MG1655 Δ*pspFABCDE::FRT*	This study
Rcs^-	MG1655 Δ*rcsDB::FRT*	This study
Psp^- Rcs^-	MG1655 Δ*pspFABCDE::FRT* Δ*rcsDB::FRT*	This study
Bae^- Cpx^- Psp^- Rcs^-	MG1655 Δ*baeSR::FRT* Δ*cpxQPRA::FRT* Δ*pspFABCDE::FRT* Δ*rcsDB::FRT*	This study

Table S5 : Plasmid list

Plasmid	Properties	Reference
pNF02-mNG	oriF *cat* λt1ter-proDp-*mNeongreen*-T7TE*luxI*A	This study
pNF06	mini F Δ*sopABC aph-2 proDp-mNeongreen*	(9)
p-*cpxP*-mNG	pNF02 ΔproDp::p*cpxP*	This study
p-*micA*-mNG	pNF02 ΔproDp::p*micA*	This study
p-*pspA*-mNG	pNF02 ΔproDp::p*pspA*	This study
p-*rcsA*-mNG	pNF02 ΔproDp::*prcsA*	This study
p-*spy*-mNG	pNF02 ΔproDp::*pspy*	This study
pKD46	$ori101^{ts}$ *bla araC* p*araB-gam-bet-exo*	(10)
pKD13	oriR6Kγ *bla FRT-aphA2-FRT*	(10)
pCP20	$ori101^{ts}$ *bla cat cI857* pL-*flp*	(10)
pET-GFP	pET-GFP plasmid contains the gfp gene under the control of an IPTG-inducible promoter	(11)

Table S6: Primer list

Name	Sequence 5'-3'
BAC-for	TACGCGTCACAGCTAACACC
BAC-rev	TGTGATACTAGAGGTGCACC
XbaI-p-cpxP-F	CCCCTCTAGATGTTTAAATACCTCCGAGGCAGAAATTACGTCATCAG
p-cpxP-RBS-BmtI-R	CCCCGCTAGCTTTCTCCTCTTTACATGACGGCAGCGGTAACTATGC
XbaI-p-micA-F	CCCCTCTAGACGGCATTTAGCCACCTCCGG
p-micA-RBS-BmtI-R	CCCCGCTAGCTTTCTCCTCTTTCGCGTCTTTCATATACTCAGACTCGCC
XbaI-p-pspA-F	CCCCTCTAGATAGCTGTTCGCCTCACCAAGTAAATTATCTTTGTATTCTGC
p-pspA-RBS-BmtI-R	CCCCGCTAGCTTTCTCCTCTTTAAGCGTTGATGTTGGCATTCACGATGTC
XbaI-p-rcsA-F	CCCCTCTAGAGCCGTTAATTGCACCTTTTTGCG
p-rcsA-RBS-BmtI-R	CGAGGCTAGCTTTCTCCTCTTATAGTCGGGTGTAACTACATAAATCC
XbaI-p-spy-F	CCCCTCTAGACCTGGTTATTTGCTTTATTTATCACCAGTCATCCGG
p-spy-RBS-BmtI-R	CCCCGCTAGCTTTCTCCTCTTTATTATGCAACAAACAGTGCAGTTAATTTACGCATATTCTATATCCTTCC
del-baeSR-Kan-f	AACGGAAGCAAATCATCTGCAATGCATTAAGCAGCAGGCAAATTGAGGATTTCCGGGGATCCGTCGACC
del-baeSR-Kan-r	GCCTGCAAATGCGAAGTTTAACTCCGCTTATACAGCGGCAACCAAATCACTGTAGGCTGGAGCTGCTTCG
del-cpx-Kan-f	ATACGCATCAAAATGTAGCTATTTCGCGGCGAAAAAGGAGCGCGCAATGATTCCGGGGATCCGTCGACC
del-cpx-Kan-r	AACAAAATAATGTCGCTAAAACTAAACGATGCGGCAGGCGTCGGCTTCCCTGTAGGCTGGAGCTGCTTCG
del-psp-Kan-f	CCGAATAAAGCATTCACGCCGCATCCGGCAAGTTGTATTGCTCAACTTCGTTCCGGGGATCCGTCGACC
del-psp-Kan-r	GGTGTGACAGAAAAAAAAACGGCGCATAAGCGCCGCTCATGGTGAATTCTTGTAGGCTGGAGCTGCTTCG
del-rcs-Kan-f	AGGATGATAAATATCACGGGAGAATAGAGAATCATCAATCAGGTAAGAGTTCCGGGGATCCGTCGACC
del-rcs-Kan-r	GCCTACAGGTGATTAGTCTTTATCTGCCGGACTTAAGGTCACTGAAGAGATGTAGGCTGGAGCTGCTTCG

Supplementary methods

Strains and plasmids construction

Deletion strains were constructed by amplification of the kanamycin resistance cassette from pKD13 using primers "del" listed in Table S4. The cassette was then transformed in recombination-competent *E. coli* MG1655 carrying the pKD46 vector. After verification of the cassette insertion, the mutation was transduced by P1 bacteriophage into wild-type MG1655 cells to prevent secondary mutations that might be caused by the lambda system. Resistance cassette was excised using the pCP20 vector.

Deletions include promoters and regulation sequences to prevent polar effects. The entire *psp* and *cpx* operons, comprising both the sensor and response regulator genes, were deleted to avoid polar effects (Cpx^-: Δ*cpxQPRA* ; Psp^-: Δ*pspFABCDE*). Since the *rcsB* promoters are located in the upstream *rcsD* ORF, both genes and their promoters were deleted (Rcs^-: Δ*rcsDB*). The BaeS sensor and BaeR regulator - encoding genes were both deleted (Bae^-: Δ*baeSR*).

Plasmids were constructed using primers listed in table S4 by standard restriction-ligation protocols. Fluorescent reporters were cloned with a strong synthetic RBS in the pNF02-mNG vector. Promoter fragments were digested with XbaI and BmtI enzymes and pNF02-mNG was cut with AvrII and BmtI.

ESR reporter validation

Inducing conditions for reporter validation were chosen as follows: 5% ethanol to activate the transcription of *micA* (6) and *spy* (2) belonging respectively to σ^E and both Bae and Cpx regulons, 2 mM zinc induces Cpx-regulated *cpxP* transcription (3), 10% isohexane induces the Psp-regulated *pspA* gene, known to be induced by organic solvents (4), and 1.4 µg/mL of polymyxin B induces Rcs-regulated *rcsA* (5).

The synthetic promoter *pro1p* (7) was used as an internal control and fluorescence values of the other reporters have been normalized on *pro1p*-mNG fluorescence accumulation in both conditions.

Supplementary references:

1. Wiegand I, Hilpert K, Hancock REW. 2008. Agar and broth dilution methods to determine the minimal inhibitory concentration (MIC) of antimicrobial substances. 2. Nat Protoc 3:163–175.

2. Bury-Moné S, Nomane Y, Reymond N, Barbet R, Jacquet E, Imbeaud S, Jacq A, Bouloc P. 2009. Global analysis of extracytoplasmic stress signaling in Escherichia coli. PLoS Genet 5:e1000651.

3. Lee LJ, Barrett JA, Poole RK. 2005. Genome-wide transcriptional response of chemostat-cultured Escherichia coli to zinc. J Bacteriol 187:1124–1134.

4. Kobayashi H, Yamamoto M, Aono R. 1998. Appearance of a stress-response protein, phage-shock protein A, in Escherichia coli exposed to hydrophobic organic solvents. Microbiology (Reading) 144 (Pt 2):353–359.

5. Mensa B, Kim YH, Choi S, Scott R, Caputo GA, DeGrado WF. 2011. Antibacterial mechanism of action of arylamide foldamers. Antimicrob Agents Chemother 55:5043–5053.

6. Udekwu KI, Wagner EGH. 2007. Sigma E controls biogenesis of the antisense RNA MicA. Nucleic Acids Res 35:1279–1288.

7. Davis JH, Rubin AJ, Sauer RT. 2011. Design, construction and characterization of a set of insulated bacterial promoters. Nucleic Acids Res 39:1131–1141.

8. Goormaghtigh F, Fraikin N, Putrinš M, Hallaert T, Hauryliuk V, Garcia-Pino A, Sjödin A, Kasvandik S, Udekwu K, Tenson T, Kaldalu N, Van Melderen L. 2018. Reassessing the Role of Type II Toxin-Antitoxin Systems in Formation of Escherichia coli Type II Persister Cells. mBio 9.

9. Jurėnas D, Fraikin N, Goormaghtigh F, De Bruyn P, Vandervelde A, Zedek S, Jové T, Charlier D, Loris R, Van Melderen L. 2021. Bistable Expression of a Toxin-Antitoxin System Located in a Cryptic Prophage of Escherichia coli O157:H7. mBio 12:e0294721.

10. Datsenko KA, Wanner BL. 2000. One-step inactivation of chromosomal genes in Escherichia coli K-12 using PCR products. Proc Natl Acad Sci USA 97:6640–6645.

11. Jõers A, Tenson T. 2016. Growth resumption from stationary phase reveals memory in Escherichia coli cultures. Scientific Reports 6:24055.
